# Perinatal Outcomes in Pregnancies Complicated by Maternal Thrombocytopenia: A Retrospective Cohort Study

**DOI:** 10.3390/jcm14134524

**Published:** 2025-06-26

**Authors:** Woo Jeng Kim, In Yang Park, Sae Kyung Choi

**Affiliations:** 1 Incheon St. Mary’s Hospital, College of Medicine, The Catholic University of Korea, Seoul 06591, Republic of Korea; yoteamo811@naver.com; 2 Seoul St. Mary’s Hospital, College of Medicine, The Catholic University of Korea, Seoul 06591, Republic of Korea; ooooobbbbb@catholic.ac.kr

**Keywords:** pregnancy, thrombocytopenia, idiopathic thrombocytopenic purpura, postpartum hemorrhage

## Abstract

**Background/Objectives**: Maternal thrombocytopenia, affecting approximately 10% of pregnancies, may be physiological (e.g., gestational thrombocytopenia) or pathological (e.g., immune thrombocytopenic purpura, aplastic anemia, preeclampsia, systemic lupus erythematosus). While gestational thrombocytopenia is typically benign, its severity and etiology may impact maternal and neonatal outcomes. This study examined the association between severe and moderate thrombocytopenia during pregnancy and perinatal outcomes, focusing on maternal hemorrhage and neonatal thrombocytopenia. **Methods**: A retrospective analysis was conducted of 182 pregnant women with thrombocytopenia who delivered at Incheon St. Mary’s Hospital and Seoul St. Mary’s Hospital between 2009 and 2019. Participants were classified into two groups: severe thrombocytopenia (platelet count <50 × 10^9^/L) and moderate thrombocytopenia (50–150 × 10^9^/L). Maternal hemorrhagic outcomes, transfusion needs, and neonatal platelet counts were evaluated. Statistical analyses were performed using univariate methods. **Results**: Severe thrombocytopenia was associated with greater blood loss during delivery, increased transfusion requirements, and elevated neonatal thrombocytopenia rates. Moderate to severe thrombocytopenia was more frequently identified in neonates delivered by mothers with immune thrombocytopenic purpura than in those delivered by mothers with gestational thrombocytopenia. **Conclusions**: Both the severity and etiology of maternal thrombocytopenia significantly affect the risk of maternal hemorrhage and neonatal thrombocytopenia. Careful prenatal assessment is essential to optimize management and reduce complications.

## 1. Introduction

Thrombocytopenia during pregnancy, commonly referred to as maternal thrombocytopenia, affects nearly one in ten pregnancies [1]. Among its various causes, gestational thrombocytopenia is the most prevalent, accounting for approximately 75% of cases [2,3]. This physiological condition results primarily from pregnancy-induced hemodilution and increased plasma volume, leading to a relative reduction in platelet concentration. Gestational thrombocytopenia is considered a benign and self-limited condition that typically presents in the second or third trimester, with platelet counts usually ranging between 100 and 150 × 10^9^/L. It is not associated with maternal or neonatal complications and typically resolves spontaneously postpartum. The diagnosis is made by exclusion, particularly ruling out immune thrombocytopenic purpura and hypertensive disorders of pregnancy [2,4,5]. However, thrombocytopenia may also arise from pathological conditions such as idiopathic thrombocytopenic purpura (ITP), fetal–neonatal alloimmune thrombocytopenia, aplastic anemia (AA), pre-eclampsia, and systemic lupus erythematosus (SLE), each involving distinct mechanisms and clinical implications [2,4,5].

Maternal thrombocytopenia presents notable clinical challenges, particularly due to the elevated risk of obstetric hemorrhage, which may necessitate blood transfusion [2,6]. This risk is heightened in cases associated with conditions such as pre-eclampsia or ITP [7,8,9]. In addition, maternal thrombocytopenia negatively impacts neonatal outcomes. Neonatal thrombocytopenia, often linked to maternal platelet disorders, is associated with serious complications, including intracranial hemorrhage [10]. Thrombocytopenia is frequently identified as one of the leading hematologic disorders in newborns in neonatal intensive care units (NICUs), particularly among preterm neonates, and warrants careful clinical monitoring [11]. Considering these maternal and neonatal risks, the management of thrombocytopenia during pregnancy requires a multidisciplinary approach that encompasses both obstetric and neonatal care.

Although several studies have examined the association between maternal thrombocytopenia and perinatal outcomes, the evidence remains inconsistent, particularly regarding the impact of thrombocytopenia severity and etiologies. For instance, gestational thrombocytopenia is often considered benign, with limited impact on maternal or neonatal morbidity [12,13]. Recognizing these gaps in the current knowledge, we aimed to evaluate the perinatal complications associated with maternal thrombocytopenia, with a particular focus on how outcomes differ according to the severity and underlying etiology of thrombocytopenia.

The objective of this study was to evaluate the association between severe thrombocytopenia and negative perinatal outcomes, in comparison to moderate thrombocytopenia. Specifically, our goal was to evaluate the frequency of maternal hemorrhagic complications and neonatal thrombocytopenia according to the severity of maternal thrombocytopenia and to analyze how different etiologies, such as gestational thrombocytopenia and ITP, impact neonatal outcomes.

## 2. Materials and Methods

### 2.1. Data Collection

This retrospective analysis was based on medical data from pregnant patients who delivered at Incheon St. Mary’s Hospital and Seoul St. Mary’s Hospital between 1 January 2009 and 31 December 2019. Eligible participants were defined as those diagnosed with thrombocytopenia during pregnancy, characterized by a platelet count below 150 × 10^9^/L, in accordance with established obstetric guidelines [2]. Patients were excluded if thrombocytopenia was diagnosed only after delivery or if they had concurrent viral infections, congenital anomalies, or multifetal pregnancies.

Maternal platelet counts were assessed at multiple time points during pregnancy. For analysis purposes, the lowest platelet count recorded between diagnosis and delivery was used. Collected maternal characteristics included age, parity, relevant medical history, gestational age at delivery, mode of delivery, and comorbid conditions potentially associated with thrombocytopenia, such as ITP, AA, pre-eclampsia, SLE, Bechet’s disease, hypothyroidism, and gestational diabetes mellitus.

### 2.2. Exposure Classification and Outcome Measures

Based on platelet counts, participants were classified into two groups: severe thrombocytopenia group (<50 × 10^9^/L) and moderate thrombocytopenia group (50–150 × 10^9^/L). This classification was used to assess outcome differences according to severity.

Maternal obstetric outcomes included estimated blood loss at delivery, the need for blood transfusion, and wound-related complications involving the episiotomy site or cesarean section scar. Neonatal outcomes encompassed the incidence and severity of thrombocytopenia, preterm birth (defined as delivery before 37 weeks of gestation), low Apgar scores, admission to the NICU, neonatal jaundice, and bruising.

Neonatal thrombocytopenia was defined as a platelet count below 150 × 10^9^/L, in accordance with established hematological standards [10], and further categorized as mild (100–150 × 10^9^/L), moderate (50–100 × 10^9^/L), or severe (<50 × 10^9^/L).

Management protocols followed institutional standards and international guidelines. All patients received active management of the third stage of labor with uterotonics (10 IU oxytocin intramuscularly), as recommended by FIGO and ACOG. In the severe thrombocytopenia group (<50 × 10^9^/L), additional measures such as preoperative intravenous immunoglobulin (IVIg) or corticosteroids (prednisone 0.25–0.5 mg/kg/day) were administered to optimize platelet counts. Prophylactic tranexamic acid (1 g IV) was also given in cases where hemorrhagic risk was anticipated.

### 2.3. Statistical Analysis

All statistical analyses were performed using SPSS software (version 26.0, IBM Corp., Armonk, NY, USA). Continuous variables were reported as the mean and standard deviation, and differences between groups were evaluated using independent samples *t*-tests. Categorical data were analyzed using Pearson’s chi-square test or Fisher’s exact test, depending on the expected frequencies. A two-tailed *p*-value of less than 0.05 was considered statistically significant. Considering the retrospective study design and the unavailability of individual-level raw data, multivariate analyses adjusted for potential confounders were not conducted. Therefore, all comparisons were limited to univariate analyses.

## 3. Results

### 3.1. Baseline Maternal Characteristics

Overall, 210 pregnant women were screened for eligibility. After excluding 28 cases due to intrauterine fetal death, multifetal gestation, or postpartum diagnosis of thrombocytopenia, total of 182 participants were analyzed, of whom 75 (41.2%) were allocated to the severe thrombocytopenia group and 107 (58.8%) to the moderate thrombocytopenia group.

The mean maternal age was 32.2 ± 4.2 years, with no significant difference between Groups A (31.9 ± 3.9 years) and B (32.4 ± 4.2 years; *p* = 0.401). Parity distribution was also comparable between the groups (*p* = 0.769).

Gestational thrombocytopenia occurred more frequently in the moderate thrombocytopenia group than in the severe thrombocytopenia group (62.3% vs. 16.0%, *p* < 0.001). Conversely, immune thrombocytopenic purpura (ITP) and aplastic anemia (AA) were more commonly observed in the severe thrombocytopenia group than in the moderate thrombocytopenia group, with respective frequencies of 68.0% vs. 27.1% (*p* < 0.001) for ITP and 18.7% vs. 2.9% (*p* < 0.001) for AA. Although the severe thrombocytopenia group delivered at a slightly earlier gestational age than the moderate thrombocytopenia group (38.0 ± 2.1 vs. 38.7 ± 1.4 weeks), both reached full term. The mode of delivery was similar between the groups, with no significant difference observed (*p* = 0.849; Table 1).

### 3.2. Obstetrical Outcomes

The severe thrombocytopenia group demonstrated a greater average estimated blood loss during delivery compared to the moderate thrombocytopenia group (528.3 ± 488 mL vs. 417.4 ± 185 mL; *p* = 0.036). Moreover, transfusion requirements were markedly elevated in the severe thrombocytopenia group, including greater average units of packed red blood cells (1.13 ± 1.63 vs. 0.27 ± 1.23; *p* < 0.001) and single-donor platelets (1.79 ± 1.62 vs. 0.49 ± 0.91; *p* < 0.001). Although transfusion rates for platelet concentrates and fresh frozen plasma were also higher in the severe thrombocytopenia group, these differences were not significant. There was no meaningful difference identified in surgical wound complications between the groups, including both episiotomy and cesarean section sites (9.3% vs. 10.3%; *p* = 0.833; Table 2).

### 3.3. Neonatal Outcomes

Mean neonatal platelet levels were lower in the severe thrombocytopenia group compared to the moderate thrombocytopenia group (218.8 ± 96.4 × 10^9^/L vs. 238.9 ± 70.8 × 10^9^/L; *p* = 0.005). Furthermore, a higher incidence of neonatal thrombocytopenia was observed in the severe thrombocytopenia group (21.3%) compared to the moderate thrombocytopenia group (5.6%, *p* = 0.024). Severe neonatal thrombocytopenia was observed in 9.3% of neonates in the severe thrombocytopenia group, markedly exceeding the 1.1% noted in the moderate thrombocytopenia group. All three neonates who required platelet transfusion belonged to the severe thrombocytopenia group.

Although the rate of preterm delivery was higher in the severe thrombocytopenia group compared to the moderate thrombocytopenia group (16.0% vs. 7.5%), this difference did not reach statistical significance. (*p* = 0.070). NICU admission, neonatal jaundice, and bruising were more common in the severe thrombocytopenia group; however, these variations were not significant (Table 3).

### 3.4. Association Between Maternal Etiology and Neonatal Thrombocytopenia

Neonatal thrombocytopenia was significantly associated with maternal ITP, occurring in 86.3% of neonates with thrombocytopenia, whereas those born to mothers without ITP had a 40.1% occurrence (*p* < 0.001). In contrast, gestational thrombocytopenia was inversely associated with neonatal thrombocytopenia, being less frequent among affected neonates (13.6%) than in those with normal platelet counts (32.6%, *p* = 0.009). No significant associations were observed for other maternal etiologies such as pre-eclampsia, SLE, or AA (Table 4).

### 3.5. Severity of Neonatal Thrombocytopenia According to Maternal Etiology

Moderate and severe neonatal thrombocytopenia were more frequently observed in neonates born to mothers with ITP compared to those born to mothers with gestational thrombocytopenia (31.8% and 31.8% vs. 9.0% and 4.5%, respectively; *p* < 0.001 and *p* = 0.008, respectively). In contrast, gestational thrombocytopenia was predominantly associated with mild or moderate forms, and rarely with severe neonatal thrombocytopenia (Table 5).

## 4. Discussion

This study investigated the relationship between maternal thrombocytopenia and perinatal outcomes, with particular attention given to the roles of thrombocytopenia severity and etiology. Our findings indicate that severe maternal thrombocytopenia is associated with increased intrapartum blood loss, higher transfusion requirements, and a greater incidence of neonatal thrombocytopenia.

Previous research has suggested that gestational thrombocytopenia generally follows a benign course and is typically associated with only mild or moderate neonatal thrombocytopenia, without substantial maternal or neonatal morbidity [13]. Likewise, low maternal platelet counts have been linked to minor mucocutaneous bleeding during delivery, but not to severe hemorrhagic complications [9]. Although these reports imply that gestational thrombocytopenia rarely leads to serious outcomes, our findings suggest that thrombocytopenia severity itself—regardless of etiology—may independently increase the risk of intrapartum hemorrhage.

Consistent with earlier studies [13], this study observed that severe neonatal thrombocytopenia was more prevalent among neonates born to mothers with immune-mediated thrombocytopenia, particularly those with ITP. Other studies have also reported associations between severe maternal thrombocytopenia and adverse neonatal outcomes, including NICU admissions, preterm births, and low birth weight [14,15]. Our findings corroborate these reports, demonstrating an elevated incidence of neonatal thrombocytopenia among infants born to mothers with severe thrombocytopenia, especially when the cause was immune-mediated.

These results emphasize the importance of considering both the severity and etiology of maternal thrombocytopenia in clinical risk assessments. Immune-mediated conditions, such as ITP or aplastic anemia, were more commonly observed in the severe thrombocytopenia group and were significantly associated with more severe neonatal outcomes. In contrast, gestational thrombocytopenia was predominantly observed in the moderate thrombocytopenia group and was typically associated with mild or moderate neonatal thrombocytopenia. This reinforces the concept that gestational thrombocytopenia rarely leads to clinically significant neonatal morbidity [10,16].

The key strength of our study is the stratification of maternal thrombocytopenia by both severity and underlying etiology. This approach enabled a more comprehensive evaluation of the condition’s impact on maternal and neonatal outcomes and provided clinically relevant insights into risk stratification. Although our findings indicate a higher risk profile in severe or immune-mediated thrombocytopenia, specific management strategies could not be assessed in this study. Further research is needed to establish evidence-based interventions.

Nonetheless, this study has certain limitations that must be acknowledged. First, the retrospective design restricts causal inferences and may be subject to measurement inaccuracies, particularly concerning estimated blood loss and transfusion documentation. Second, variations in institutional clinical practices, such as the timing of neonatal platelet count measurements and thresholds for transfusion, may have influenced the outcome assessment. Future prospective studies are warranted to confirm these findings and develop standardized management protocols for thrombocytopenia during pregnancy. Third, this study is limited by a relatively small sample size. Moreover, our findings specifically address risk differentials within thrombocytopenic pregnancies rather than absolute risks compared to the general population. The relatively low number of cases over a 10-year period reflects the strict inclusion and exclusion criteria applied. We excluded women with multifetal pregnancies, postpartum diagnoses, certain comorbidities, and those who delivered elsewhere, which may have contributed to the lower observed rate compared to the general obstetric population. To mitigate this, we standardized outcomes and adhered to established hematologic thresholds. Future prospective studies with larger cohorts and more comprehensive datasets could validate these findings while controlling for confounders such as regional treatment protocols and should include multivariate analyses to better account for confounding variables.

## 5. Conclusions

An association was observed between maternal thrombocytopenia and an elevated risk of adverse perinatal events, particularly when thrombocytopenia was severe or immune-mediated. Intrapartum hemorrhagic events and transfusion requirements were significantly more frequent among women with severe thrombocytopenia. Moreover, both the incidence and the clinical severity of neonatal thrombocytopenia were significantly higher in neonates born to mothers with immune-mediated conditions, particularly idiopathic thrombocytopenic purpura.

These findings underscore the importance of thorough antenatal evaluation of thrombocytopenia in pregnancy, with careful consideration of both platelet count and underlying etiology. These findings highlight the importance of recognizing that women with severe or immune-mediated thrombocytopenia, and their neonates, are at a higher risk of adverse outcomes. Although no specific preventive strategies are currently available, awareness of this elevated risk may support more informed antenatal and perinatal monitoring. A multidisciplinary approach including obstetricians, hematologists, and neonatologists may help optimize clinical outcomes and reduce maternal and neonatal morbidity.

## Figures and Tables

**Table 1:** The obstetrical characteristics of the study populations.

Characteristic	Severe Thrombocytopenia Group	Moderate Thrombocytopenia Group	*p*-Value
N (%)	75 (41.2%)	107 (58.8%)	
Maternal age (years)	31.9 ± 3.9	32.4 ± 4.2	0.401
**Parity**			0.769
Primigravida (%)	48 (64.0%)	63 (58.9%)	
Multigravida (%)	27 (36.0%)	44 (41.1%)	
History of abortion (%)	64 (84.0%)	49 (46.2%)	<0.001
**Cause of thrombocytopenia**			
Gestational thrombocytopenia (%)	12 (16.0%)	66 (62.3%)	<0.001
ITP (%)	51 (68.0%)	29 (27.1%)	<0.001
Aplastic anemia (%)	14 (18.7%)	3 (2.9%)	<0.001
Pre-eclampsia (%)	3 (4.0%)	5 (4.8%)	1.000
SLE (%)	3 (4.0%)	2 (1.9%)	0.651
Bechet’s disease (%)	1 (1.3%)	3 (2.9%)	0.642
GDM (%)	4 (5.3%)	6 (5.8%)	1.000
Hypothyroidism (%)	3 (4.0%)	9 (8.7%)	0.364
Gestation at delivery (weeks)	38.0 ± 2.1	38.7 ± 1.4	0.011
**Mode of delivery**			0.849
Vaginal delivery (%)	48 (64.0%)	67 (62.6%)	
Cesarean section (%)	27 (36.0%)	40 (37.4%)	

**Abbreviations**: N, number of patients; ITP, idiopathic thrombocytopenic purpura; SLE, systemic lupus erythematosus; GDM, gestational diabetes mellitus.

**Table 2:** Obstetrical outcomes related to hemorrhage.

Outcome	Severe Thrombocytopenia Group	Moderate Thrombocytopenia Group	*p*-Value
Blood loss at delivery (mL)	528.31 ± 488	417.43 ± 185	0.036
**Transfusion (mean units)**			
PRC	1.13 ± 1.63	0.27 ± 1.23	<0.001
SDP	1.79 ± 1.62	0.49 ± 0.91	<0.001
PC	1.61 ± 3.63	0.82 ± 2.95	0.122
FFP	0.17 ± 0.86	0.07 ± 0.44	0.320
Complications associated with episiotomy site or C/S scar (%)	7 (9.3%)	11 (10.3%)	0.833

**Abbreviations:** PRC, packed red blood cell; SDP, single-donor platelet; PC, platelet concentrate; FFP, fresh frozen plasma; C/S, cesarean section.

**Table 3:** Neonatal outcomes according to the severity of maternal thrombocytopenia.

Outcome	Severe Thrombocytopenia Group	Moderate Thrombocytopenia Group	*p*-Value
Neonatal platelet count (x10^9^/L)	218.81 ± 96.39	238.89 ± 70.82	0.005
Neonatal thrombocytopenia (%)	16 (21.3%)	6 (5.6%)	0.024
**Severity of neonatal thrombocytopenia** *			0.063
Mild (%)	3 (4.0%)	2 (1.9%)	
Moderate (%)	6 (8.1%)	3 (2.9%)	
Severe (%)	7 (9.3%)	1 (1.1%)	
Preterm delivery (%)	12 (16.0%)	8 (7.5%)	0.070
NICU care (%)	18 (26.5%)	14 (14.7%)	0.672
Neonatal jaundice (%)	28 (41.2%)	36 (37.9%)	0.162
Neonatal bruising (%)	4 (5.3%)	1 (1.1%)	0.162
**Apgar score < 7**			
5 min (%)	7.83 ± 1.75	8.08 ± 1.23	0.246
10 min (%)	8.96 ± 1.65	9.29 ± 0.92	0.088

**Abbreviations:** NICU, neonatal intensive care unit. * **Severity classification**: mild (platelet count: 100–150 × 10^9^/L); moderate (platelet count: 50–100 × 10^9^/L); severe (platelet count < 50 × 10^9^/L).

**Table 4:** Association between maternal etiology and neonatal thrombocytopenia.

Maternal Etiology	Neonates with Normal Platelet Counts	Neonates with Thrombocytopenia	*p*-Value
Gestational thrombocytopenia (%)	62 (32.6%)	3 (13.6%)	0.009
ITP (%)	57 (40.1%)	19 (86.3%)	<0.001
AA (%)	16 (11.2%)	1 (4.5%)	0.474
Pre-eclampsia (%)	8 (5.6%)	0 (0.0%)	0.389
SLE (%)	5 (3.5%)	0 (0.0%)	1.000
Bechet’s disease (%)	4 (2.8%)	0 (0.0%)	1.000
GDM (%)	8 (5.6%)	0 (0.0%)	0.600
Hypothyroidism (%)	9 (6.4%)	3 (13.6%)	0.211

**Abbreviations:** ITP, idiopathic thrombocytopenic purpura; AA, aplastic anemia; SLE, systemic lupus erythematosus; GDM, gestational diabetes mellitus.

**Table 5:** Severity of neonatal thrombocytopenia according to maternal etiology.

Cause of Maternal Thrombocytopenia	Severity of Neonatal Thrombocytopenia			*p*-Value
	**Mild**	**Moderate**	**Severe**	
Gestational thrombocytopenia (%)	0 (0.0%)	2 (9.0%)	1 (4.5%)	0.008
ITP (%)	5 (22.7%)	7 (31.8%)	7 (31.8%)	<0.001
Aplastic anemia (%)	0 (0.0%)	0 (0.0%)	1 (4.5%)	0.521
Hypothyroidism (%)	2 (9.0%)	0 (0.0%)	1 (4.5%)	0.564

**Abbreviations:** ITP, idiopathic thrombocytopenic purpura. **Severity classification:** mild (100–150 × 10^9^/L); moderate (50–100 × 10^9^/L); severe (<50 × 10^9^/L).

## Data Availability

The data supporting the findings of this study are available from the corresponding author upon request.

## Abbreviations

AA	Aplastic anemia
ITP	Idiopathic thrombocytopenic purpura
SLE	Systemic lupus erythematosus
NICU	Neonatal intensive care unit
GDM	Gestational diabetes mellitus
PRC	Packed red cell
SDP	Single-donor platelet
